# Vortioxetine Improves Depressive Symptoms and Cognition in Parkinson’s Disease Patients with Major Depression: An Open-Label Prospective Study

**DOI:** 10.3390/brainsci12111466

**Published:** 2022-10-29

**Authors:** Diego Santos García, Maria Gema Alonso Losada, Icíar Cimas Hernando, Iria Cabo López, Rosa Yáñez Baña, Ruben Alonso Redondo, Jose Manuel Paz González, Carlos Cores Bartolomé, Maria José Feal Painceiras, Maria Cristina Íñiguez Alvarado, Carmen Labandeira, Iago García Díaz

**Affiliations:** 1 Department of Neurology, Hospital Universitario de A Coruña (HUAC), CHUAC (Complejo Hospitalario Universitario de A Coruña), C/As Xubias 84, 15006 A Coruña, Spain; 2 CHUVI (Complejo Hospitalario Universitario de Vigo), 36312 Vigo, Spain; 3 Hospital Povisa, 36211 Vigo, Spain; 4 CHOP (Complejo Hospitalario de Pontevedra), 36071 Pontevedra, Spain; 5 CHUO (Complejo Hospitalario Universitario de Orense), 32005 Ourense, Spain; 6 Hospital Lucus Augusti, 27003 Lugo, Spain

**Keywords:** depression, effectiveness, open-label study, Parkinson’s disease, vortioxetine

## Abstract

Depression is frequent in Parkinson’s disease (PD) patients, but the evidence for many antidepressant agents to treat it in PD is insufficient. The aim of the present prospective open-label single-arm study (VOPARK, an open-label study of the effectiveness and safety of VOrtioxetine in PARKinson’s disease patients with depression) was to analyze the effectiveness of vortioxetine on depressive symptoms in PD patients with major depression. The primary efficacy outcome was the change from baseline (VB) at the end of the observational period (12 weeks ± 14 days; V12w) in the 17-item Hamilton Depression Rating Scale (HAM-D17) total score. At VB, all patients had a HAM-D17 total score ≥16. A total of 30 patients (age 66.23 ± 10.27; 73.3% males) were included between February 2021 (first patient, 12/FEB/21) and March 2022 (last patient, 14/MAR/22). At 12 weeks, 27 patients completed the follow-up (90%). The total HAM-D17 total score was reduced by 52.7% (from 21.5 ± 4.75 at VB to 10.44 ± 7.54 at V12w; Cohen’s effect size = −2.5; *p* < 0.0001) and the response and remission rates were 50% and 43.3%, respectively. Apathy (Apathy Scale; *p* < 0.0001), cognition (PD-Cognitive Rating Scale; *p* = 0.007), fatigue (Fatigue Severity Scale; *p* = 0.014), and quality of life (PDQ-39 (*p* = 0.001) and EUROHIS-QOL8 (*p* < 0.0001)) improved at 3 weeks as well. A total of 11 adverse events in 10 patients (33.3%) were reported, one of which was severe (vomiting related to vortioxetine with full recovery after drug withdrawal). Vortioxetine was safe and well tolerated and improved depressive symptoms and other non-motor symptoms in PD patients.

## 1. Introduction

Depression has been strongly associated with Parkinson’s disease (PD), with previous studies estimating the prevalence rate to be between 2.7 to 90% [1]. A systematic review found the weighted prevalence of major depressive disorder to be 17% in PD patients, that of minor depression to be 22%, and that of dysthymia to be 13% [2]. Clinically significant depressive symptoms were present in 35% of patients. Depression is a key determinant of a reduced quality of life (QoL) in PD [3]. Depression has also been associated with sleep disturbances, fatigue, cognitive dysfunction, and decreased functional ability with impairment of activities of daily living (ADL) [4]. Despite the magnitude and impact of depression in PD, there is a shortage of properly conducted large, randomized, clinical trials of antidepressants in PD. The studies reported are limited by their sample size, use of different scales, and heterogeneous patient populations. The main groups of drugs that have been evaluated are dopamine agonists, tricyclic antidepressants (TCAs), and selective serotonergic and norepinephrine reuptake inhibitors (SSRIs and SNRIs) [5]. There is a lack of evidence for recommendations about how to treat depression in PD patients, with evidence being insufficient for many antidepressant agents [6].

In September 2013, vortioxetine was approved by the FDA for the treatment of major depressive disorder in adults in United States. Vortioxetine has different mechanisms of action; it is a SERT inhibitor; antagonist of 5-HT3, 5-HT7, 5-HTID; partial agonist of 5-HT1B; and a full agonist of 5-HT1A [7]. Moreover, vortioxetine does not appear to interact significantly with the norepinephrine transporters or dopamine transporters, but its administration has been shown to increase extracellular levels of norepinephrine, dopamine, and non-monoamine neurotransmitters including acetylcholine. These effects are also thought to be related to the interaction between vortioxetine and various serotonin receptors [8]. Vortioxetine has been demonstrated to be efficacious, safe, and well-tolerated in many randomized, double-blind, placebo controlled, and/or active treatment-referenced clinical trials [9]. Moreover, vortioxetine seems to be safe when it is administered to elderly patients and, interestingly, it could improve cognitive function compared to other antidepressants [10,11,12,13,14]. Other symptoms such as anxiety, pain, or sleep have been reported to improve with vortioxetine as well [15,16,17]. In PD, some non-motor symptoms (NMS) can be related to decreased levels of dopamine (apathy, anhedonia, cognitive problems, etc.) as well as other neurotransmitters such as serotonin (depression, etc.), noradrenaline (orthostatic hypotension, pain, etc.), or acetylcholine (dementia, etc.) [18]. For all this, vortioxetine could be a useful antidepressant agent with interesting possibilities in the treatment of depression in PD patients. However, there is limited evidence on the use of vortioxetine in patients with PD [19,20,21,22]. Some data suggest that vortioxetine may improve depression severity without significant worsening of motor symptoms and with a remarkable safety profile in PD patients [19,20].

The aim of the present prospective open-label single-arm study (VOPARK, an open-label study of the effectiveness and safety of VOrtioxetine in PARKinson’s disease patients with depression) was to analyze the effectiveness of vortioxetine on depressive symptoms in PD patients with major depression (dPD). Secondary objectives were to analyze the effectiveness of vortioxetine on apathy, cognitive function, fatigue, QoL, and functional capacity for ADL in dPD patients, as well as its safety and tolerability.

## 2. Materials and Methods

VOPARK is a single-country (Spain), multicenter, observational (phase IV), prospective open-label follow-up study. Eight neurology sites from Galicia (Spain) that deal with dPD participated. A total of 40 consecutive dPD patients were expected to be included in the study, corresponding to 5 patients/site. The inclusion criteria were: (1) a diagnosis of Parkinson’s disease according to the United Kingdom Parkinson’s Disease Society Brain Bank criteria [23]; (2) a diagnosis of major depression according to Diagnostic and Statistical Manual of Mental Health Disorders, 5th Edition (DSM-5) criteria [24]; (3) a 17-item Hamilton Depression Rating Scale (HAM-D_17_) score ≥ 16 [25,26]; (4) undergoing stable dopaminergic treatment and no expectations of dose or drug changes in the next 3 months; (5) no dementia criteria [27]; (6) older than 40 years old; and (7) wishing to voluntarily participate. The exclusion criteria were: (1) contraindication to being treated with vortioxetine according to the product data; (2) to receive at baseline evaluation or to have received up to 15 days before the baseline evaluation a SSRI and/or SNRI (tricyclics such as amitriptyline and heterocyclics such as mirtazapine or trazodone antidepressant agents were allowed); (3) being pregnant and/or breastfeeding; (4) an incapacity to complete the questionnaires adequately; (5) other disabling concomitant neurological diseases (stroke, severe head trauma, neurodegenerative disease, etc.); (6) other severe and disabling concomitant non-neurological diseases (oncological, autoimmune, etc.); (7) expected impossibility of long-term follow-up; or (8) patients who were already participating in a clinical trial and/or other type of study.

The study visits included 3 visits: (1) VB (baseline); (2) V4w (4 weeks ± 7 days; telephonic visit); and (3) V12w (12 weeks ± 14 days; end of the Observational Period). At Baseline (VB), after providing written informed consent to participate in the study, subjects completed an assessment that included motor symptoms (Hoehn and Yahr (H&Y) [28]; Unified Parkinson’s Disease Rating Scale (UPDRS) part III and part IV [29]), mood (HAM-D_17_) [25]), apathy (Apathy Scale (AS) [30]), cognition (Parkinson’s Disease Cognitive Rating Scale (PD-CRS) [31]), fatigue (Fatigue Severity Scale (FSS) [32], health-related QoL (the 39-item Parkinson’s disease Questionnaire (PDQ-39) [33]) and global QoL (EUROHIS-QOL8 item index [34]), and autonomy for ADL (Schwab and England Activities of Daily Living Scale (ADLS) [35]. In all the scales/questionnaires, a higher score indicates a more severe affectation—with the exception of the PD-CRS, ADLS, and EUROHIS-QOL8, in which the opposite is the case (Supplementary Material Table S1). The V4w was conducted by phone to check the safety and tolerability of the drug and also to adjust the dose according to the neurologist’s criteria. The Patient and doctor Global Impression of Change (CGI-C) scale [36] together with the same scales administered in the baseline visit—except for the H&Y and UPDRS-IV—were assessed at V12w. All visits were conducted with the patient during the ON-state (after taking his/her medication for PD). Information on sociodemographic aspects, factors related to PD, comorbidities, and treatments was collected.

The primary objective of the study was to analyze the effectiveness of vortioxetine on depressive symptoms at 12 weeks in dPD patients. A reduced version with 17 items [25] of the original version of the 21-item Hamilton Depression Rating Scale [37] was administered, like the rest of the scales, by a neurologist. All the neurologists who participated in the study of each center were experts on PD/movement disorders. According to the major depression DSM-5 criteria, the individual has to be experiencing five or more symptoms during the same 2-week period—with at least one of the symptoms being either (1) a depressed mood or (2) a loss of interest or pleasure—including: (1) depressed mood most of the day, nearly every day; (2) markedly diminished interest or pleasure in all, or almost all, activities most of the day, nearly every day; (3) significant weight loss when not dieting or weight gain, or a decrease or increase in appetite nearly every day; (4) a slowing down of thought and a reduction in physical movement (observable by others, not merely subjective feelings of restlessness or being slowed down); (5) fatigue or loss of energy nearly every day; (6) feelings of worthlessness or excessive or inappropriate guilt nearly every day; (7) a diminished ability to think or concentrate, or indecisiveness, nearly every day; or (8) recurrent thoughts of death, recurrent suicidal ideation without a specific plan, a suicide attempt, or a specific plan for committing suicide. To receive a diagnosis of depression (which was given by the neurologist), these symptoms had to cause the individual clinically significant distress or impairment in social, occupational, or other important areas of functioning. The symptoms also had to not be the result of substance abuse or another medical condition.

Secondary objectives included: (1) an analysis of the effectiveness of vortioxetine on apathy (AS), cognitive function (PD-CRS), and fatigue (FSS); (2) an analysis of the effectiveness of vortioxetine on QoL (health-related (PDQ-39) and global (EUROHIS-QOL8 item index)) and functional capacity for ADL (ADLS); (3) an assessment of the clinical global impression of change according to the patient (PGI-C) and the clinician (CGI-C); and (4) an analysis of the safety and security of vortioxetine in PD patients.

Vortioxetine was administered as a once-daily 10 mg pill, with the possibility of increasing the dose at the neurologist’s indication. However, in patients aged 65 years or older, according to the product data sheet or other considerations made by the neurologist, the first dose should be 5 mg. A dose adjustment could be made after the VB visit as well (from 5–10 mg to 15 and/or 20 mg/day). During the follow-up, any medications other than vortioxetine could not be changed (regimen, doses, etc.)—except if the neurologist considered these changes absolutely necessary. All changes, including PD and non-PD-related medications and the levodopa equivalent daily dose (LEDD), [38] were recorded.

### 2.1. Data Analysis

Data were processed using SPSS (Statistical Package for Social Sciences) 20.0 for Windows. Continuous variables were expressed as the mean ± SD or median and quartiles, depending on whether they were normally distributed. Each domain of the PDQ-39 was expressed as a percentage: (score/total score) × 100. Relationships between variables were evaluated using the Student’s t-test, the Mann–Whitney U test, or the Spearman’s or Pearson’s correlation coefficient, as appropriate (the distribution of the variables was verified by a one-sample Kolmogorov–Smirnov test). Correlations were considered weak for coefficient values ≤0.29, moderate for values between 0.30 and 0.59, and strong for values ≥0.60.

The primary efficacy outcome was the change from baseline (VB) to the end of the observational period (12 weeks) in the HAM-D_17_ total score. According to the HAM-D_17_ total score (ranging from 0 to 52), patients were classified at VB and V12w as not depressed (0–7 points); with mild/minor depression (8–13 points); with moderate depression (14–18 points); with severe depression (19–22 points); or with very severe depression (>23 points). Moreover, other aspects were analyzed at V12w: the completion rate (% of patients receiving vortioxetine); response rate (% of patients with a 50% or greater reduction in the HAM-D_17_ total score); and remission rate (% of patients without criteria for depression (HAM-D_17_ total score, 0–7)).

Changes from the VB at V12w in the total scores of the UPDRS-III, AS, PD-CRS, FSS, PDQ-39SI, EUROHIS-QOL8 item index, and ADLS were the secondary efficacy outcome variables. Analyses on efficacy variables were performed with the ITT data set (all subjects who received at least 1 pill of vortioxetine and had a baseline and treatment observation for the primary efficacy outcome measure). A paired-sample t-test or Wilcoxon’s rank sum, McNemar, or marginal homogeneity tests were performed when appropriate for testing changes from the baseline. Cohen’s d formula was applied for measuring the effect size, which was considered to be absent, <0.2; small, 0.2–<0.5; moderate, 0.5–<0.8; large, 0.8–1.3; or very large, ≥1.3. Values of *p* < 0.05 were considered significant.

The safety data set consists of all subjects for whom the study device was initiated. Safety analyses were assessed by adverse events (AEs). All AEs were coded using the current version of the Medical Dictionary for Regulatory Activities (MedDRA). The number and percentage of subjects with treatment-emergent AEs—ordered according to the MedDRA system organ class and preferred term, their severity, and their relationship to the study treatment, as assessed by the investigator—was provided for all subjects.

Standard protocol approvals, registrations, and patient consent were obtained.

For this study, we received approval from the Comité de Ética de la Investigación Clínica de Galicia, Spain (2020/129; 31/MAR/2020). Written informed consent from all participants in this study was obtained before the beginning of the study. VOPARK was classified by the AEMPS (Agencia Española del Medicamento y Productos Sanitarios) as a Post-authorization Prospective Follow-up study, with the code DSG-VOR-2020-01.

### 2.2. Data Availability

The protocol and the statistical analysis plan are available on request. De-identified participant data are not available for legal and ethical reasons.

## 3. Results

A total of 30 patients (aged 66.23 ± 10.27; 73.3% males) were included between February 2021 (first patient, 12/FEB/21) and March 2022 (last patient, 14/MAR/22). The percentage of patients recruited with respect to the expected sample size was 75% (30/40). Two centers did not recruit any patients and two patients failed the screening (neither met the criteria for major depression). Data on sociodemographic aspects, comorbidities, antiparkinsonian drugs, and other therapies are shown in Table 1. The mean time from symptom onset in PD was 4.16 ± 3.11 years. All patients were receiving an antiparkinsonian treatment (levodopa 96.7%; MAO-B inhibitor 76.7%; dopamine agonist 60%; COMT inhibitor 23.3%) and three were undertaking a second-line therapy (one deep brain stimulation; one subcutaneous apomorphine infusion; one enteral levodopa/carbidopa infusion). Benzodiazepines, antidepressant agents (allowed by the protocol), and analgesic drugs were taken by 43.3%, 20%,and 20% of the patients, respectively. The mean LEDD was 765.25 ± 477.63 (ranging from 100 to 2150 mg).

**Figure 1:**
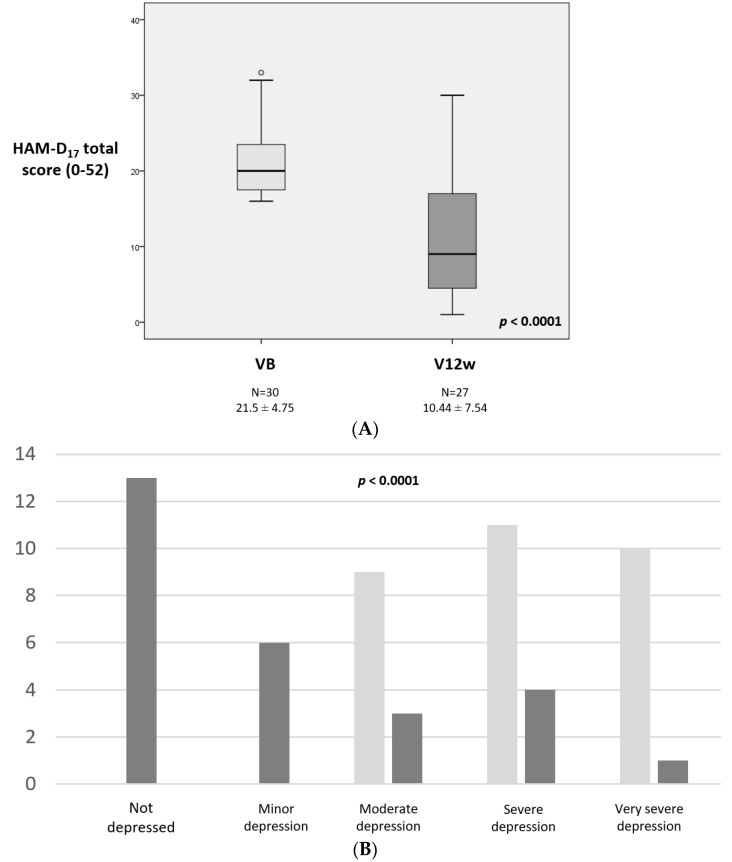
(**A**) HAM-D17 total score at VB (baseline) and V12w (12 weeks ± 14 days); *p* < 0.0001. (**B**) Number of cases with different type of depression at VB vs V12w: not depressed, HAM-D17 0–7; mild/minor depression, HAM-D17 8–13; moderate depression, HAM-D17 14–18; severe depression, HAM-D17 19–22; very severe depression, HAM-D17 > 23 (*p* < 0.0001). Data are presented as box plots, with the box representing the median and the two middle quartiles (25–75%). *p* values were computed using the Wilcoxon signed-rank test (**A**) and the marginal homogeneity test (**B**). Mild outliers (O) are data points that are more extreme than Q1–1.5. HAM-D17—17-item Hamilton Depression Rating Scale.

At baseline (VB), the mean HAM-D_17_ total score was 21.5 ± 4.75 (ranging from 16 to 33) and 70% of the patients had severe or very severe depression (Figure 1B). At 12 weeks, 27 patients completed the follow-up (90%). The mean vortioxetine starting dose was 5.5 ± 1.52 mg/day (5 mg, N = 27; 10 mg, N = 3) whereas the final dose at V12w was 9.61 ± 3.13 (5 mg, N = 5; 10 mg, N = 20; 15 mg, N = 1; 20 mg, N = 1). The total HAM-D_17_ total score was reduced by 52.7% (from 21.5 ± 4.75 at VB to 10.44 ± 7.54 at V12w; Cohen’s effect size = −2.5; *p* < 0.0001; Table 2 and Figure 1A) and the response and remission rates were 50% and 43.3%, respectively. Only five patients (18.5%) had severe or very severe depression, whereas 19 out of 27 (70.3%) had no depression or minor depression at the end of follow-up (Figure 1B). By scale items, the effect of improvement was very large on “Depressed mood” (Cohen’s effect size = −2.23; *p* < 0.0001) and “Work and activities” (Cohen’s effect size = −1.51; *p* < 0.0001; Supplementary Material Table S2).

**Figure 2:**
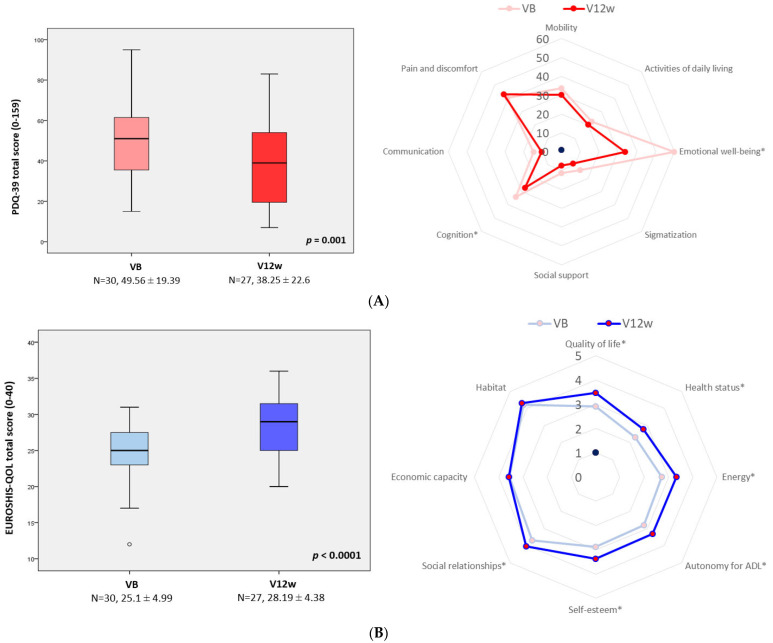
(**A**) PDQ-39 total score at VB (baseline) and V12w (12 weeks ± 14 days); *p* = 0.001. Mean score on each domain of the PDQ-39 at VB and V12 expressed as the Summary Index (range 0–100). The difference between both visits was significant for PDQ-39D3 (Emotional well-being; *p* < 0.0001) and PDQ-39D6 (Cognition; *p* = 0.033). (**B**) EUROSHIS-QOL8 total score at VB (baseline) and V12w (12 weeks ± 14 days); *p* < 0.0001. Mean score on each domain (range 0–5) of the EUROHIS-QOL8 at VB and V12w. The difference between both visits was significant (* *p* < 0.05; Table 2) for all domains except EUROSHIS-QOL8D7 (Economic capacity) and EUROSHIS-QOL8D8 (Habitat). Data are presented as box plots, with the box representing the median and the two middle quartiles (25–75%). *p* values were computed using the Wilcoxon signed-rank test. Mild outliers (O) are data points that are more extreme than Q1–1.5. PDQ-39—the 39-item Parkinson’s disease Questionnaire.

**Figure 3:**
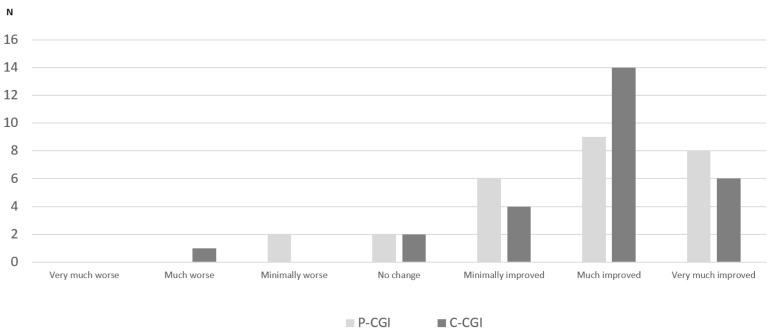
Clinical global impression of changes according to the patient’s (PGI-C) and clinician’s (CGI-C) opinion (N = 27). Total number of patients accorded to each category are shown (*y*-axis).

A significant change from VB to V12w, indicating an improvement, was observed with other scales (AS, PD-CRS, FSS, PDQ-39, EUROHIS-QOL8; Table 2). No significant changes were observed in the UPDRS-III score (*p* = 0.483). Regarding cognitive-specific aspects, only a significant improvement in “Delayed verbal memory” was observed (from 4.4 ± 2.67 at VB vs 4.96 ± 2.54; Cohen’s effect size = +0.54; *p* = 0.047), but a trend of significance in the fronto-subcortical (*p* = 0.104) and cortical-posterior (*p* = 0.098) domains was detected (Table 2). Both health-related and global QoL improved after the 12-week follow-up: PDQ-39, from 49.56 ± 19.39 to 28.25 ± 22.6 (Cohen’s effect size = −0.78; *p* = 0.001); EUROHIS-QOL8, from 25.1 ± 4.99 to 38.25 ± 28.19 ± 4.38 (Cohen’s effect size = +1.35; *p* < 0.0001) (Figure 2). By domains, a significant improvement was detected in “Emotional well-being” (Cohen’s effect size = −1.28; *p* < 0.0001) and “Cognition” (Cohen’s effect size = −0.6; *p* = 0.033), as well as in all domains of the EUROHIS-QOL8 except “Economic capacity” and “Habitat” (Table 2 and Figure 2). After the end follow-up (change from VB to V12w), the improvement detected in mood (HAM-D_17_ total score) correlated with the improvement observed in apathy (AS; r = 0.465; *p* = 0.015), fatigue (FSS; r = 0.497; *p* = 0.008), and the health-related QoL (PDQ39; r = 0.406; *p* = 0.036) (Table 3). At V12w, 23 patients out of 27 (85.2%) felt better regarding the PGI-C: eight very much improved; nine much improved; six minimally improved; two no changes; and two minimally worse. Similar results were recorded with the CGI-C (Figure 3).

A total of 11 adverse events in 10 patients (33.3%) were reported—one of which was severe (vomiting related to vortioxetine, with full recovery 6 days later after drug withdrawal; Table 4). Gastrointestinal symptoms (nausea, N = 5; vomiting, N = 1; 20%) and dizziness (6.6%) were the most frequent. A full recovery was observed in 9 out of 11 (81.8%) adverse events. Three patients discontinued due to different reasons: personal decision of the patient (N = 1); changes in treatment by a psychiatrist; or an SAE (severe adverse event; N = 1).

## 4. Discussion

The present study observed that PD patients with major depression improved in terms of their depressive symptoms 3 months after starting with vortioxetine. Moreover, vortioxetine was safe and well-tolerated, and other aspects such as cognitive function, apathy, fatigue, and QoL improved after the 3-month follow-up as well. No motor impairments were detected. Importantly, this is the first prospective, published study specifically designed to analyze the effects of vortioxetine on depressive symptoms and other NMSs in PD.

Depression occurs in around 35% of patients with PD and is often persistent [39]. Many cross-sectional and prospective longitudinal studies have demonstrated that depression is a key factor impacting not only patients’ QoL, but also principal caregivers’ QoL too [3,40,41,42]. The underlying mechanisms of depression in PD are not known in detail, but changes in brain structure, signaling by neurotransmitters, and levels of inflammatory and neurotrophic factors are all suggested to contribute to its development [39,43]. Psychosocial factors, sleep problems, and pain could also have roles in depression [39]. Changes in dopaminergic, noradrenergic, and serotonergic systems in patients with PD might help to explain the incidence of depression in PD patients—so dopaminergic drugs and antidepressants with serotonergic and/or noradrenergic effects could be used to treat depression in PD. However, pramipexole [44] and nortriptyline [45] are the only agents that have shown antidepressant effects in placebo-controlled clinical trials in patients with PD, and evidence regarding recommendations on how to treat depression in PD is poor [6]. Moreover, PD patients very frequently develop other NMSs (apathy, fatigue, pain, cognitive impairment, etc.) that can be related to the diversity of pathways and neurotransmitters (dopamine, serotonin, noradrenaline, acetylcholine, etc.) involved in PD [46]; in this context, vortioxetine—an antidepressant agent with a unique pharmacological profile and a multimodal mechanism of action, with the possibility of increasing levels in the brain of five neurotransmitters (serotonin, dopamine, histamine, noradrenaline, and acetylcholine) [47]—could be a good option for trying to improve not only depressive symptoms, but also other NMSs in patients with PD. Despite this, the evidence on the use of vortioxetine in patients with PD is very scarce [19,20,21,22]—this was the justification for proposing the VOPARK study.

In the VOPARK study, the mean reduction in the HAM-D_17_ total score was 53% (−10.9 points), the response rate was 50%, and complete remission was observed in 43.3% of patients at the 3-month follow-up. Many randomized, placebo-controlled trials have demonstrated the efficacy of vortioxetine for the treatment of major depressive disorder in adults [48,49,50]. Our results are similar to those reported in patients randomized to vortioxetine in double-blind trials using the HAM-D, with reductions ranging from −11.08 points at 8 weeks with 5 mg/day vortioxetine to −16.23 points at 8 weeks with 10 mg/day vortioxetine [51,52,53,54]. Open-label extension studies with vortioxetine have reported response and remission rates of up to 94% and 83%, respectively [55]. However, the evidence on the use of vortioxetine in patients with PD is very poor, with only five entries in PubMed with the search terms “Parkinson” and “vortioxetine” (September 2022). Russo et al. [19] observed in 150 PD patients treated with vortioxetine—with a mean score at baseline on the HAM-D of 18 points—a reduction on the scale of eight points with 10 mg and seven points with 20 mg, without any severe side effects. However, the data are not published [19]. Miliukhina (article written in Russian) observed in 150 PD patients with mild to moderate depression treated with vortioxetine, a significant improvement in depressive symptoms and anxiety after 12 weeks of treatment—with good tolerability and without motor function impairment [20]. A phase IV trial on the “Tolerability, Safety and Efficacy of Vortioxetine” (VorDe-PD; NCT04301492) in patients with a diagnosis of sustained depression and a HAM-D_17_ ≥ 14 is ongoing.

In our study, patients improved not only in terms of their depressive symptoms, but also in terms of cognitive function, apathy, and fatigue. Previous studies on elderly patients with major depression have detected that vortioxetine could have a multi-domain beneficial effect on cognitive performance—including executive function, attention/speed of processing, and memory [51,56,57]. Changes in EEG (decreased theta power and increased beta power) in patients with major depression treated with vortioxetine have been reported [58]. A beneficial effect on cognition has been observed in elderly patients with Alzheimer’s disease (AD) as well [11], although a recent randomized, double-blind, placebo-controlled study in 100 AD patients was negative [59]. The efficacy of vortioxetine on cognition has been mainly linked to its action on synaptic serotonin levels via the inhibition of the serotonin (5-HT) transporter (SERT), but its cognition-enhancing properties are hypothesized to also be mediated by other mechanisms such as the release of acetylcholine [60]. Regarding the “dual syndrome” hypothesis of cognitive impairment in PD, there are independent contributions of both dopaminergic denervation on fronto-striatal cognition—including executive impairment—and cholinergic denervation on visuospatial and other attentional impairments in PD [61]. The small sample size could explain why the effect on both cognitive sub-scores in our cohort was not significant, although a significant effect on delayed verbal memory and a clear trend in the posterior cortical sub-score together with the lack of correlation between cognitive function improvement and mood improvement might suggest a possible effect of vortioxetine related to cholinergic function. On the contrary, improvements in apathy and fatigue correlated with improvements in depressive symptoms. Although the pathophysiology of fatigue and apathy in PD is clearly multifactorial, in a proportion of PD patients, these symptoms are associated with depression, dopaminergic depletion in the mesocorticolimbic structures, and disruption of the prefrontal cortex–basal ganglia axis [62]—so, improving depressive symptoms with vortioxetine might produce improvement in apathy and fatigue. A previous meta-analysis found that compared with a placebo, vortioxetine improved physical symptoms in 2105 adults with major depression [63]. Our results agree with these findings—with improvement in insomnia, anxiety, and gastrointestinal and general somatic symptoms according to the HAM-D_17_. Symptoms such as anxiety, pain, or sleep have previously been reported to improve with vortioxetine [15,16,17]. Moreover, the improvement in depressive symptoms and other NMSs in PD patients in our study was accompanied by an improvement in the patient’s health-related and global QoL. Data from previous studies conducted in adults with major depression demonstrates that vortioxetine improves the patient’s QoL after 6 to 8 weeks of treatment [64].

Vortioxetine was not only effective but also safe and well-tolerated, with the drug maintenance rate at 6 months being very high (90%). In line with the literature [65], gastrointestinal symptoms (nausea and/or vomiting) were the most frequent adverse event, being present in one out of five patients. According to an analysis of data pooled from 11 randomized, placebo-controlled acute treatment studies (3018 patients treated with vortioxetine; six studies included venlafaxine as an active reference) and five open-label, long-term extension studies (2457 patients treated for up to 52 weeks with vortioxetine), the most common treatment-emergent adverse events associated with vortioxetine were nausea (20.9–31.2%) and vomiting (2.9–6.5%)—the incidence of which reached a plateau at 15 mg/day 20 [66]. This complication must be taken into account in PD patients, who are sensitive to the effects of dopaminergic medication. Our recommendation to prevent this complication is to take vortioxetine after eating on a full stomach or before going to bed, and always starting with 5 mg per day for a few days. In PD patients, previous, very limited data suggest that vortioxetine is safe and well-tolerated [19,20] and that it does not seem to worsen motor symptoms [19]. In fact, a very recent study conducted in rats reported that vortioxetine ameliorated motor impairments in rotenone-induced PD via the targeting of TLR-2-mediated neuroinflammation [67].

Our study has some important limitations. The most important is related to the study design itself (open-label study), and since there is not a comparative arm with a placebo, the results should be interpreted with caution. Second, the sample size is small (30 patients out of the 40 initially proposed) and it is possible that the changes observed in some variables (e.g., cognitive sub-scores) were not significant due to this. In fact, due to different problems (i.e., the COVID-19 pandemic and the identification of patients with PD and major depression not taking antidepressant medication), the study was closed before reaching the initially planned sample size (N = 40). Third, the data were collected from patients with PD and major depression, so it is necessary to be cautious when extrapolating the results to PD patients with depressive symptoms without major depression (minor depression, subclinical depression, dysthymia, etc.). On the other hand, this is the first prospective study specifically designed to assess the effect of vortioxetine on depressive symptoms and other NMSs in PD patients with depression. Despite these limitations, the results presented here are novel and are of great interest as there is a lack of knowledge on what effects vortioxetine can have over mood and many other symptoms in PD patients.

In conclusion, this study observed that PD patients improved in terms of their depressive symptoms and other related NMSs (cognition, apathy, and fatigue) 3 months after starting treatment with vortioxetine, with good tolerability. Based on these results and despite some limitations, these findings suggest that vortioxetine could be a good option for treating depression in patients with PD in clinical practice.

## Figures and Tables

**Table 1:** Data about sociodemographic aspects, comorbidities, antiparkinsonian drugs and other therapies at baseline (N = 30).

Age	66.23 ± 10.27 (48–83)	Family cases of depression (%)	33.3
Gender (males) (%)	73.3	Family cases of PD (%)	26.7
Race (%)			
Caucasian	100	Time from symptoms onset	4.16 ± 3.11 (0.33–11)
Other	0		
		Motor fluctuations (%)	60
Civil status (%):		Dyskinesia (%)	23.3
Married	53.3		
Widowed	23.3	Treatment for PD (%):	
Single	10	Levodopa	96.7
Divorced	10	MAO-B inhibitor	76.7
Other	3.4	COMT inhibitor	23.3
		Dopamine agonist	60
Living style (%)		Amantadine	6.7
With the partner	56.7		
Alone	20	L-dopa daily dose (mg)	505.71 ± 392.56 (0–1910)
With a son/daughter	20	LEDD (mg)	765.25 ± 477.63 (100–2150)
Other	3.3		
		Other treatments (%):	
Habitat (%):		Amitriptiline	6.6
Rural (<5000)	10	Trazodone	10
Semiurban (5000–20,000)	26.7	Mirtazapine	3.3
Urban (>20,000)	63.3	Benzodiazepine	43.3
		Antipsychotic	3.3
Comorbidities (%):		Analgesic	20
Arterial hypertension	40		
Diabetes mellitus	6.7	Number of anti-PD drugs	2.86 ± 1.3 (1–6)
Dyslipemia	36.7	Number of non-PD drugs	2.82 ± 2.8 (0–9)
Hiperuricemia	3.3	Total number of drugs	5.68 ± 2.96 (1–13)
Cardiomyopathy	3.3	Number of pills for PD	4.87 ± 2.26 (1–9.5)
Cardiac arrhythmia	3.3	Number of pills for other cause	2.62 ± 2.49 (0–8.5)
Smoking	6.7	Total number of pills	7.5 ± 2.68 (3–13.75)
Alcohol consumption	0		

The results represent % or mean ± SD (range). COMT, catechol-O-methyltransferase; LEDD, levodopa equivalent daily dose; MAO-B, Monoamine oxidase-B; PD, Parkinson’s disease.

**Table 2:** Change in the HAM-D_17_ total score and other scales of the study from VB (baseline; N = 30) to V12w (12 weeks ± 14 days; N = 27).

	VB	V12w	Cohen’s *d*	∆VB–V12w	*p*
MOTOR ASSESSMENT					
H&Y-ON	2 (1.75–2)	N.A.	N. A	N.A.	N.A.
UPDRS-III-ON	23.1 ± 9.85 (9–51)	21.63 ± 8.28 (7–39)	−0.21	−6.90%	0.483
UPDRS−IV	2.53 ± 2.04	N.A.	N.A.	N.A.	N.A.
NON MOTOR ASSESSMENT					
HAM-D_17_	21.5 ± 4.75 (16–33)	10.44 ± 7.54 (1–30)	−2.5	−52.70%	<0.0001
AS	17.6 ± 6.54 (1–31)	11.29 ± 7.18 (1–26)	−1.3	−35.10%	<0.0001
PD-CRS	80.66 ± 19.14 (29–116)	86.81 ± 20.45 (38–127)	0.8	7.94%	0.007
PD-CRS FS sub-score	54.17 ± 18.19	59 ± 18.96	0.39	8.90%	0.104
Immediate verbal memory	7.3 ± 2.03 (4–12)	7.85 ± 2.14 (4–12)	0.46	7.50%	0.091
Sustained attention	7.37 ± 3.21 (0–10)	8.33 ± 2.07 (2–10)	0.49	13%	0.094
Working memory	5.9 ± 2.67 (0–9)	6.19 ± 2.2 (0–19)	0.08	4.90%	0.946
Clock drawing	8.57 ± 2.3 (1–10)	9 ± 1.54 (5–10)	0.16	5%	0.711
Delayed verbal memory	4.4 ± 2.67 (0–11)	4.96 ± 2.54 (0–10)	0.54	12.70%	0.047
Alternating verbal fluency	9.67 ± 4.22 (2–17)	10.7 ± 4.71 (2–20)	0.48	10.60%	0.114
Action verbal fluency	12.53 ± 4.68 (5–24)	13.07 ± 5.61 (6–27)	0.25	4.30%	0.654
PD-CRS PC sub-score	26.5 ± 8.94	27.81 ± 7.06	0.44	4.90%	0.098
Confrontation naming	15.57 ± 4.98 (7–24)	17.48 ± 3.78 (8–26)	0.46	12.30%	0.067
Clock copy	9.2 ± 2.14 (1–10)	9.37 ± 1.36 (4–10)	0.22	1.80%	0.566
FSS	38.7 ± 18.49 (9–76)	29.04 ± 16.3 (9–60)	−0.77	−27.90%	0.014
QOL AND AUTONOMY					
PDQ-39	49.56 ± 19.39 (15–95)	38.25 ± 22.6 (7–83)	−0.78	−23.80%	0.001
Mobility	33.83 ± 22 (0–35)	30.37 ± 24.27 (0–35)	−0.26	−10.20%	0.109
Activities of daily living	22.64 ± 18.94 (0–18)	20.22 ± 18.37 (0–16)	−0.23	−10.70%	0.273
Emotional well-being	59.72 ± 24.05 (1–24)	33.95 ± 24.23 (0–21)	−1.28	−43.20%	<0.0001
Stigmatization	13.96 ± 18.18 (0–10)	8.8 ± 13 (0–6)	−0.46	−36.90%	0.092
Social support	11.39 ± 18.88 (0–8)	7.41 ± 13.73 (0–6)	−0.38	−35.20%	0.143
Cognition	34.17 ± 26.19 (0–15)	27.31 ± 22.14 (0–13)	−0.6	−20.10%	0.033
Communication	14.72 ± 16.47 (0–6)	10.49 ± 15.77 (0–6)	−0.48	−28.70%	0.069
Pain and discomfort	40.56 ± 21.52 (0–10)	43.21 ± 26.85 (0–12)	0.2	6.50%	0.583
EUROHIS-QOL8	25.1 ± 4.99 (12–38)	28.19 ± 4.38 (20–36)	1.35	12.30%	<0.0001
Quality of life	2.93 ± 0.94 (1–4)	3.48 ± 0.7 (2–4)	0.9	18.70%	0.004
Health status	2.3 ± 0.87 (1–4)	2.78 ± 0.93 (1–4)	0.71	20.80%	0.02
Energy	2.73 ± 0.98 (1–5)	3.33 ± 0.92 (1–5)	0.96	21.90%	0.002
Autonomy for ADL	2.8 ± 1.03 (1–5)	3.3 ± 0.95 (2–5)	1.01	17.80%	0.002
Self-esteem	2.87 ± 1.04 (1–5)	3.37 ± 1 (1–5)	0.86	17.40%	0.004
Social relationships	3.7 ± 0.75 (1–5)	4.04 ± 0.51 (3–5)	0.61	9.10%	0.025
Economic capacity	3.57 ± 0.72 (2–5)	3.59 ± 0.84 (1–5)	0.24	0.50%	0.356
Habitat	4.2 ± 0.61 (3–5)	4.3 ± 0.61 (3–5)	0.51	2.30%	0.059
ADLS	82.66 ± 11.72 (50–100)	84.81 ± 11.22 (50–100)	0.32	2.60%	0.227
Functional dependency (%)	23.3	14.8	N.A.	N.A.	0.687

*p* values were computed using the Wilcoxon signed-rank or Mc Nemar test. The results represent mean ± SD (range), median [p25, p75] or %. Each domain of the PDQ-39 was expressed as a percentage: (score/total score) x 100. Cohen’s d formula was applied for measuring the effect size. It was considered: ignored, <0.2; small, 0.2 –<0.5; moderate, 0.5 –<0.8; large, 0.8–1.3; very large, ≥1.3. N.A., not applicable. ADLS, Schwab & England Activities of Daily Living Scale; AS, Apathy Scale; FSS, Fatigue Severity Scale; HAM-D_17_, 17-item Hamilton Depression Rating Scale score; H&Y: Hoenh & Yahr; PD-CRS, Parkinson’s Disease Cognitive Rating Scale; PDQ-39, 39-item Parkinson’s Disease Quality of Life Questionnaire; QoL, quality of life; UPDRS, Unified Parkinson’s Disease Rating Scale.

**Table 3:** Correlation between the change in the HAM-D_17_ total score from VB to V12w (∆V12w–VB) and the change in the score in other scales from VB to V12w.

∆V12W–VB	HAM-D_17_	*p*
AS	0.465	0.015
PD-CRS	−0.087	0.667
PC-CRS FS sub-score	−0.221	0.268
PD-CRS PC sub-score	0.01	0.961
FSS	0.497	0.008
PDQ39	0.406	0.036
EUROHIS-QOL8	−0.235	0.238
ADLS	0.103	0.609

Pearson correlation coefficient was applied. ADLS, Schwab & England Activities of Daily Living Scale; AS, Apathy Scale; FSS, Fatigue Severity Scale; HAM-D_17_, 17-item Hamilton Depression Rating Scale score; H&Y: Hoenh & Yahr; PD-CRS, Parkinson’s Disease Cognitive Rating Scale; PDQ-39, 39-item Parkinson’s Disease Quality of Life Questionnaire; QoL, quality of life.

**Table 4:** Adverse events in patients from VB to V12w.

	N
Total AEs, N	11
Nausea	5
Dizziness	2
Vomiting	1
Headache	1
Helicobacter pylori infection	1
COVID-19 disease	1
Patients with at least one AE, N (%)	10 (33.3)
At least possibly related AEs, N	8
Definitely related	2
Probably related	4
Possibly related	2
Unrelated	3
Patients with at least possibly * related to vortioxetine AEs, N (%)	7 (23.3)
Severity, N	
Mild	9
Moderate	1
Severe	1
Total SAEs, N	1
Vomiting	
Patients with al least one SAE, N (%)	1 (3.3)
At least possibly * related to vortioxetine SAEs, N	1
Patients with at least possibly related to vortioxetine SAEs, N (%)	1 (3.3)
Patients with at least one AE leading to discontinuation, N (%)	1 (3.3)
Patients with at least one possibly * related to vortioxetine AE leading to discontinuation N (%)	1 (3.3)
Action taken with the AE, N	
Drug withdraw	1
Dose reduction	1
None	9
Deaths, N (%)	0 (0%)
Outcome of the EA, N	
Fully recovered	9
Improvement (not fully recovered)	1
Unknown	1

* Considered “possibly”, “probably” or “definitely” related to treatment (vortioxetine). AE, adverse event; SAE, serious adverse event.

## Data Availability

The protocol and the statistical analysis plan are available on request. Deidentified participant data are not available for legal and ethical reasons.

## Supplementary Materials

The following supporting information can be downloaded at: https://www.mdpi.com/article/10.3390/brainsci12111466/s1, Table S1: Description of each of the scales used in this study; Table S2: Change in each item of the HAM-D17 from baseline to final visit.

## Abbreviations

ADLS: Schwab and England Activities of Daily Living Scale; AS, Apathy Scale; FSS, Fatigue Severity Scale; HAM-D17, 17-item Hamilton Depression Rating Scale score; H&Y: Hoenh and Yahr; NMS, non-motor symptoms; PD, Parkinson’s disease; PD-CRS, Parkinson’s Disease Cognitive Rating Scale; PDQ-39, 39-item Parkinson’s Disease Quality of Life Questionnaire; QoL, quality of life; UPDRS, Unified Parkinson’s Disease Rating Scale.
