# Supplementary material for: Vortioxetine Improves Depressive Symptoms and Cognition in Parkinson’s Disease Patients with Major Depression: An Open-Label Prospective Study
Source: Brain Sci. 2022 Oct 29;12(11):1466. doi: 10.3390/brainsci12111466 (PMC9688230; doi:10.3390/brainsci12111466)
Supplement: Supplementary file 1 [file brainsci-12-01466-s001.zip › brainsci-1945048-supplementary.pdf]

Supplementary Materials

Table S1. Description of each of the scales used in this study.

17-item Hamilton Depression Rating Scale (HAM-D_{17}) [25]
The patient is rated by a clinician on 17 items scored either on a 3-point or 5-point Likert-type scale. For the 17-item version, a score of 0–7 is considered to be normal.

Hoehn and Yahr [28]
The Hoehn and Yahr Scale is used to measure how Parkinson's symptoms progress and the level of disability. Originally published in 1967 in the journal Neurology by Melvin Yahr and Margaret Hoehn, it included stages 1 to 5: 1, unilateral involvement only usually with minimal or no functional disability; 2, bilateral or midline involvement without impairment of balance; 3, bilateral disease, mild to moderate disability with impaired postural reflexes but physically independent; 4, severe disability but still able to walk or stand unassisted; 5, confinement to bed or wheelchair unless aided.

Unified Parkinson's Disease Rating Scale part III (UPDRS-III) [29]
The UPDRS-III is assessed by clinical observation. In part III different aspects of motor symptoms are assessed: speech, facial expression, tremor, rigidity, bradykinesia, posture, postural reflexes and gait. UPDRS-III score is from 0 (no signs of parkinsonism) to 108 (maximum affectation possible total score).

Apathy Scale (AS) [30]
The AS includes 14 questions assessing the domains of lack of motivation relative to the individual's previous level of functioning, lack of effort to perform every day activities, dependency on others to structure activities, lack of interest in learning new things or in new experiences, lack of concern about one's personal problems, unchanging or flat affect, and lack of emotional response to positive or negative personal events. For questions 1-8, the scoring system is the following: not at all = 3 points; slightly = 2 points; some = 1 point, a lot = 0 point. For questions 9-14: the scoring system is the following: not at all = 0 points; slightly = 1 point; some = 2 points; a lot = 3 points. The total score goes from 0 (no apathy) to 72 (maximum level of apathy).

Parkinson's Disease Cognitive Rating Scale (PD-CRS) [31]
The PD-CRS is a validated scale for assessing the cognitive status of the patients with PD. The total score is the sum of the fronto-subcortical score (item 1, immediate free recall verbal memory; item 3, sustained attention; item 4, working memory; item 5, unprompted drawing of a clock; item 7, delayed free recall verbal memory; item 8, alternating verbal fluency; item 9, action verbal fluency) and the posterior cortical score (item 2, confrontation naming; item 6, copy drawing of a clock).

Fatigue Severity Scale (FSS) [32]
The FSS is a questionnaire containing nine statements that attempt to explore severity of fatigue symptoms. The subject is asked to read each statement and circle a number from 1 to 7, depending on how appropriate they felt the statement applied to them over the preceding week. A low value indicates that the statement is not very appropriate whereas a high value indicates agreement. The scoring is done by calculating the average response to the questions (adding up all the answers and dividing by nine).

39-item Parkinson's disease Quality of Life Questionnaire (PD-39) [33]

The PDQ-39 is a questionnaire that assesses the patient's health-related quality of life. There are 39 items grouped into 8 domains: (1) Mobility (items 1 to 10); (2) Activities of daily living (items 11 to 16); (3) Emotional well-being (items 17 to 22); (4) Stigmatization (items 23 to 26); (5) Social support (items 27 to 29); (6) Cognition (items 30 to 33); (7) Communication (items 34 to 36); (8) Pain and discomfort (items 37 and 39). For each item, the score may range from 0 (never) to 4 (always). The symptoms refer to the 4 weeks prior to assessment. The total score ranges from 0 to 156 (maximum, 39 x 4) but is usually expressed as a percentage (PDQ-39SI). Every domain can also be expressed as a percentage to be able to make comparisons.

EUROHIS-QOL8 item index (EUROHIS-QOL8) [34]

The EUROHIS-QOL is an 8-item quality of life questionnaire (quality of life, health status, energy, autonomy in activities of daily living, self-esteem, social relationships, economic capacity and habitat) derived from the WHOQOL-100 and the WHOQOL-BREF. For each item, the score ranges from 0 (not at all) to 5 (completely). The total score is expressed as the mean of the individual scores or as the sum (from 0 to 30). A higher score will indicate, a prior, a higher quality of life.

Schwab & England Activities of Daily Living Scale (ADLS) [35]

In ADLS, the patient must indicate the degree of autonomy for carrying out activities of daily living, on a scale of 0 (bed-ridden, vegetative functions such as swallowing, bladder and bowel functions are not functioning) to 100 (completely independent, essentially normal). A score ≥ 80 indicates that the patient is completely independent.

Clinical global improvement (CGI) at 3 months [36]

This is a 7-point scale asking about changes in the assessed condition compared to the baseline status. The options are: 0 = Not assessed; 1 = Very much improved; 2 = Much improved; 3 = Minimally improved; 4 = No change; 5 = Minimally worse; 6 = Much worse; 7 = Very much worse. The opinion of the patient (P-CGI) and/or the evaluator (physician) (C-CGI) can be considered.

Table S2. Change in each item of the HAM-D_{17} from VB (baseline; N = 30) to V12w (12 weeks ± 14 days; N = 27).

	**VB**	**V12w**	**Cohen´s *d***	**ΔVB–V12w**	***p***
Depressed mood	3.16 ± 0.83 (1-4)	1.11 ± 2.21 (0-4)	-2.23	-64.8%	<0.0001
Feelings of guilt	1.46 ± 1.31 (0-4)	0.41 ± 0.69 (0-2)	-1.1	-71.9%	0.001
Suicide	0.86 ± 1.11 (0-3)	0.18 ± 0.62 (0-3)	-0.79	-79.1%	0.010
Insomnia: early in the night	0.56 ± 0.72 (0-2)	0.33 ± 0.67 (0-2)	-0.69	-41.1%	0.020
Insomnia: middle of the night	0.93 ± 0.58 (0-2)	0.48 ± 0.57 (0-2)	-1.13	-48.3%	0.001
Insomnia: early hours of the morning	1.13 ± 0.77 (0-2)	0.7 ± 0.77 (0-2)	-0.89	-38.1%	0.005
Work and activities	2.63 ± 1.06 (0-4)	1 ± 1.1 (0-4)	-1.51	-61.9%	<0.0001
Retardation	1.13 ± 1.04 (0-3)	0.85 ± 1.02 (0-3)	-0.41	-24.7%	0.130
Agitation	0.63 ± 1.15 (0-4)	0.29 ± 0.72 (0-3)	-0.56	-53.9%	0.047
Anxiety (psychotic)	2.46 ± 1.13 (0-4)	1.18 ± 1.33 (0-4)	-1.22	-52%	0.001
Anxiety (somatic)	1.76 ± 1.16 (0-4)	1 ± 0.91 (0-3)	-0.92	-43.2%	0.004
Gastrointestinal somatic symptoms	0.53 ± 0.73 (0-4)	0.18 ± 0.48 (0-3)	-0.65	-66%	0.026
General somatic symptoms	1.63 ± 0.61 (0-2)	1 ± 0.87 (0-2)	-0.79	-38.6%	0.006
Genital symptoms	0.56 ± 0.81 (0-2)	0.33 ± 0.62 (0-2)	-0.24	-41.1%	0.351
Hypocondrosis	1.46 ± 1.16 (0-3)	1.11 ± 1.05 (0-3)	-0.33	-23.9%	0.234
Loss of weigh	0.13 ± 0.43 (0-2)	0.03 ± 0.19 (0-1)	-0.36	-76.9%	0.180
Insight	0.4 ± 0.67 (0-2)	0.22 ± 0.57 (0-2)	-0.24	-45%	0.473

p values were computed using the Wilcoxon signed-rank. The results represent mean ± SD (range). Cohen´s d formula was applied for measuring the effect size. It was considered: ignored, <0.2; small, 0.2 –<0.5; moderate, 0.5 –<0.8; large, 0.8–1.3; very large, ≥1.3. HAM-D_{17}, 17-item Hamilton Depression Rating Scale score.
